# Correlates of poor glycemic control in patients with type 2 diabetes: results of a nationwide survey in Brazil

**DOI:** 10.1186/1758-5996-7-S1-A55

**Published:** 2015-11-11

**Authors:** Sandra S Moreira, Carine de Sousa Andrade, Carlos Antonio de Souza Teles Santos, Raimundo Celestino S Neves, Guilherme de Sousa Ribeiro, Edson Duarte Moreira

**Affiliations:** 1 Escola de Nutrição/Universidade Federal da Bahia, Salvador, Brazil

## Background

Diabetes is a metabolic disease that increases the risk of cardiovascular and microvascular disease, particularly when glycemic blood levels are not well controlled. Poor glycemic control is commonly observed in as much as 60% to 80% of patients with diabetes, regardless of advances in diabetes care. The objective of this study was to identify correlates of poor glycemic control in a large multicenter survey of Brazilian patients with type 2 diabetes.

## Materials and methods

A cross-sectional study was conducted in a consecutive sample of patients aged 18 yrs. or older with type 2 diabetes, attending health centers located in ten large cities in Brazil. Data on socio-demographics, treatment, and adherence to treatment were obtained by trained interviewers, using a standardized questionnaire. A peripheral blood sample was collected and HbA1c levels were measured by high-performance liquid chromatography at a central laboratory. Patients with HbA1c >7% were considered to have inadequate glycemic control. HbA1c was described by mean and standard deviation (SD). Bivariate linear regression analysis was performed to identify patients characteristics correlated with serum levels of HbA1c. Statistical significance was set at p<0.05.

## Results

Overall, 5,692 patients with type 2 diabetes were surveyed (mean age, 61 yrs.; female 66.5%). Mean level of HbA1c was 8.6% (SD=2.3) and the prevalence of inadequate glycemic control was 73%. Poor glycemic control was associated with young age, black or mixed race, low educational attainment and diabetes duration. We also observed that irregular medical visits in the last 12 months, self-perception of poor diet, insulin or oral hypoglycemic adherence were correlated with inadequate glycemic control. Participation in a diabetes health education program was associated with improved glycemic control.

## Conclusions

The majority of patients with type 2 diabetes in Brazil has inadequate glycemic control and is at risk for the development of cardiovascular and microvascular disease. Our data may help the development of public health programs to improve glycemic control in this population.

